# One Health Genomic Study of Human and Animal *Klebsiella pneumoniae* Isolated at Diagnostic Laboratories on a Small Caribbean Island

**DOI:** 10.3390/antibiotics11010042

**Published:** 2021-12-30

**Authors:** Patrick Butaye, Marc Stegger, Arshnee Moodley, Peter Damborg, Andrea Williams, Iona Halliday-Simmonds, Luca Guardabassi

**Affiliations:** 1 Department of Biomedical Sciences, Ross University School of Veterinary Medicine, Basseterre, Saint Kitts and Nevis; IHalliday-Simmonds@rossvet.edu.kn; 2 Department of Pathobiology, Pharmacology and Zoological Medicine, Faculty of Veterinary Medicine, Ghent University, 9820 Merelbeke, Belgium; 3 Department of Bacteria, Parasites, and Fungi, Statens Serum Institute, 2300 Copenhagen, Denmark; mtg@ssi.dk; 4 Antimicrobial Resistance and Infectious Diseases (AMRID) Research Laboratory, Murdoch University, Murdoch 6150, Australia; 5 Department of Veterinary and Animal Sciences, Faculty of Health and Medical Sciences, University of Copenhagen, 1165 Frederiksberg, Denmark; asm@sund.ku.dk (A.M.); pedam@sund.ku.dk (P.D.); 6 Joseph N France General Hospital, St Kitts, Brumaire, Saint Kitts and Nevis; aamw58@yahoo.com

**Keywords:** *Klebsiella pneumoniae*, One Health, vervet, animal, whole genome sequencing

## Abstract

*Klebsiella pneumoniae* causes a variety of infections in both humans and animals. In this study, we characterised the genomes of human and animal isolates from two diagnostic laboratories on St. Kitts, a small Caribbean island inhabited by a large population of vervet monkeys. In view of the increased chances of direct or indirect contact with humans and other animal species, we used the One Health approach to assess transmission of *K. pneumoniae* across host species by sequencing 82 presumptive *K. pneumoniae* clinical isolates from humans (n = 51), vervets (n = 21), horses (n = 5), dogs (n = 4) and a cat (n = 1). Whole genome sequencing (WGS) was carried out using Illumina technology. De novo assembly was performed in CLC Genomics Workbench v.11.0. Single nucleotide polymorphisms were detected using NASP followed by phylogenetic analysis using IQ-TREE. Virulence and antimicrobial resistance gene contents were analysed using the Kleborate and CGE pipelines. WGS-based analysis showed that 72 isolates were *K. pneumoniae* sensu stricto and five *K. quasipneumoniae* and five *K. variicola*. *K. pneumoniae* isolates belonged to 35 sequence types (ST), three of which were occasionally shared between humans and animals: ST23, ST37 and ST307. The ST23 strains from vervets formed a separate cluster amongst publicly available sequenced ST23 strains, indicating the presence of a specific vervet sublineage. Animal strains harbored fewer resistance genes and displayed distinct virulence traits that appeared to be host-specific in vervet isolates. Our results show that *K. pneumoniae* infections on this Caribbean island are usually caused by host-specific lineages.

## 1. Introduction

*Klebsiella pneumoniae* is an important nosocomial pathogen that often displays multidrug resistance, including resistance to last choice antimicrobials such as carbapenems. While there are many publications describing the epidemiology, pathogenicity and antimicrobial resistance of this pathogen in humans [1], little information is available on clinical isolates from animals [2,3,4,5]. *K. pneumoniae* has been associated with infections in horses [6,7,8], cats and dogs [9], monkeys [10,11] and cattle [12,13]. The types of infections seen in these animal species, except for mastitis in cattle, are similar to those observed in humans, mainly urinary tract infections (UTI) and septicemia [9]. Comparing the genomes of strains of different host origin can be used as one means of estimating the risk of across-host transfer, including zoonotic potential. Carbapenem resistance has been documented in clinical isolates from companion animals but also, in some cases, from livestock [14]. A growing number of reports on the occurrence of OXA-48 and other types of carbapemenases in bacteria isolated from companion animals have been published worldwide [14,15,16]. However, few data are available on the genetic relatedness of animal and human isolates. Recent studies suggest that *K. pneumoniae* might be transmitted between dogs and owners within the same household [17], and that human and canine strains causing UTI are genetically related [9,17,18,19].

In this study, we characterized the genomes of clinical *K. pneumoniae* isolates obtained at the diagnostic laboratories of a hospital and a veterinary school on the small Caribbean island of St. Kitts. The objective was to assess the risk of zoonotic transmission within this confined geographical area. The island is an ideal place to perform this type of study since it is a small island of about 174 km^2^ with an estimated population of approximately 57,000 people, and an even larger population of African green monkeys or vervets (*Chlorocebus sabaeus*) that were introduced during the 17th century on ships running the slave trade from Africa. Based on available data from the main hospital in the Federation of St. Kitts and Nevis, which deals with most of the patients in the country, the incidence of *Klebsiella* infections in humans is about 80 infections annually. Nevertheless, UTIs may also be treated by primary practitioners and, as such, the data may be an underestimation. Our study was conceived after two previous studies showed that vervets on the island suffer fatal infections caused by hypermucoid *K. pneumoniae* [20] and are a reservoir of this pathogen, with carriage rates of approximately 40% and 20% in captive and wild-caught individuals, respectively [11]. The true level of infections in vervets cannot be estimated as most animals are living in the wild and no data are available. Available local isolates obtained from companion animals (dog, cat, and horse) were included in the study to study whether there could be another potential source of *K. pneumoniae* infections on the island, as these animals are in contact with both vervets and humans as well as each other.

## 2. Results

### 2.1. Species Identification

**Figure 1:**
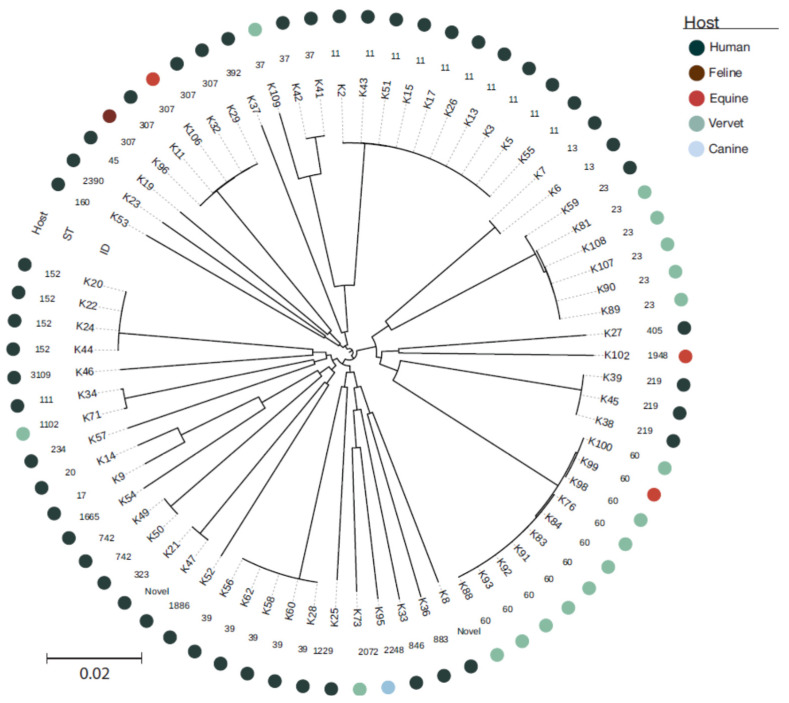
Host origin, sequence type and SNP-based phylogenetic analysis of all isolates of the study. A figure including analysis of both core genome and accessory genome can be found in Supplementary File S2.

Among the 82 isolates identified as *K. pneumoniae* by matrix-assisted laser desorption/ionization time-of-flight mass spectrometry (MALDI-TOF MS), 72 were *K. pneumoniae* sensu stricto (50 human, 17 vervet, three equine, one canine and one feline), five *Klebsiella quasipneumoniae* subspecies *quasipneumoniae* (one human, two canine and two equine) and four *Klebsiella variicola* subspecies *variicola* (three vervet and one canine) based on Whole Genome Sequence (WGS) analysis. Multilocus sequence typing (MLST) of the 72 *K. pneumoniae* sensu stricto isolates led to identification of 35 STs, 27 of which were detected amongst human isolates. The two most prevalent lineages among human isolates were sequence type (ST) ST11 (10/50 human isolates) and ST39 (5/50) (Figure 1). Vervet isolates displayed a limited number of STs with ST60 (9/17) and ST23 (5/17) dominating (Figure 1). STs for the remaining *K. pneumoniae* isolates are displayed in Figure 1. MLST analysis of the five *K. variicola* subspecies *variicola* isolates showed that they were all different STs: ST697, ST1708-DLV (double locus variant), ST1791-SLV (single locus variant), ST209-SLV and ST549-SLV. Similarly, all five *K. quasipneumoniae* subspecies’ *quasipneumoniae* isolates belonged to distinct STs: ST1539, ST2638, ST1077-DLV, ST2637-SLV and ST338-SLV.

**Figure 2:**
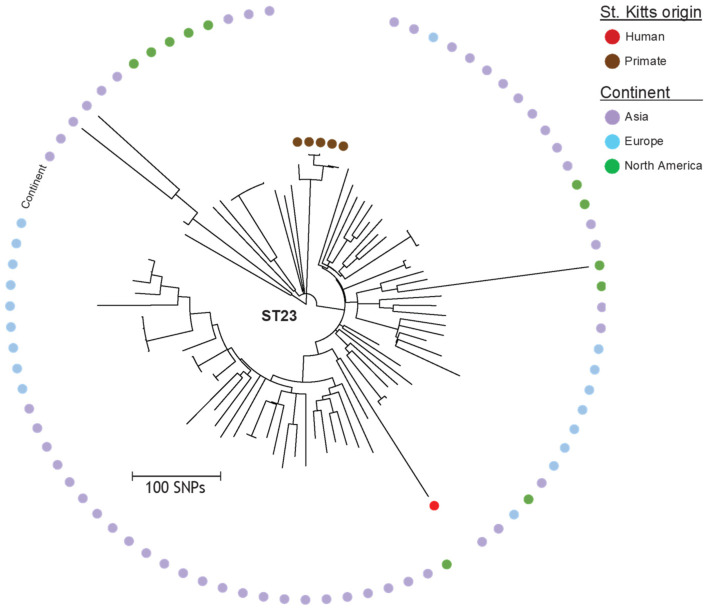
Detailed SNP-based phylogeny of the five vervet and the single human ST23 *K. pneumoniae* isolates from this study as well as 80 internationally available human ST23 isolates from NCBI’s RefSeq database. Color labels for isolates of this study represent host (vervet or human), whereas color labels for other isolates represent continent of origin (Asia, Europe or North America).

Potential exchange of *K. pneumoniae* between host species was limited to four STs: ST23 (five vervets and one human), ST307 (one horse, one cat and four humans), ST37 (one vervet and one human) and ST60 (one horse and nine vervets). However, significant differences were observed in the accessory genome of isolates from different hosts belonging to the same ST (Supplementary File S1). A more detailed investigation of the vervet ST23 isolates indicated that they formed a specific clade among the 80 available ST23 genomes in NCBI’s Reference Sequence Database (RefSeq) with data on host and geography (Figure 2).

### 2.2. Resistance Gene Content

Host-specific differences were also observed in resistance gene content. Human *K. pneumoniae* isolates generally carried a larger number of resistance genes compared to animal isolates (Supplementary File S1). Most (37/50) human isolates carried at least one acquired resistance gene, whereas the vast majority (19/22) of animal isolates did not carry any. The only three animal isolates harboring acquired resistance genes were two ST307 isolates from a horse and a cat, and one ST37 isolate from a vervet. The equine and feline ST307 isolates shared several resistance genes [*bla*_CTX-M-15_, *bla*_OXA-1_, *aph(3′)-Id*, *aph(6)*, *aac(3)-IIa*, *aac(6′)-Ib-cr*, *qnrB1*, *sul2*, *dfrA14* and *catB3*] and had mutations at *gyrA* and *parC* conferring fluoroquinolone resistance, but the equine isolate additionally carried *tet*(A) and *bla*_TEM1b_, whereas the feline isolate carried *bla*_TEM-206_. In contrast, none of the three human ST307 isolates carried *bla*_OXA-1_ and all of them contained *tet*(A). Similarly, the resistance gene profile of the ST37 isolate from vervet [*bla*_TEM-1b_, *aph(3′)-Id*, *aph(6)*, *aac(3)-IId*, *aadA2*, *sul2*, *dfrA12* and *tet*(B)] differed from that of the human ST37 isolate in many resistance genes [*aadA2*, *sul1*, *sul2*, *dfrA15*, *catA2*, *cmlA1* and *tet*(D)] (Supplementary File S1).

Apart from these host-specific trends, WGS analysis revealed the presence of *bla*_CTX-M-15_ (n = 27) encoding extended-spectrum β-lactamase (ESBL); the other β-lactamases detected were *bla*_TEM-1b_ (n = 24), *bla*_TEM-206_ (n = 1) and *bla*_OXA-1_ (n = 11), apart from the chromosomal SHV-, LEN- or OKP. Five fosfomycin resistance genes (all contained *oqxA* and *oqxB* as well as a *fos* variant: *fosA* (n = 77), *fosA5* (n = 2) and *fosA7* (n = 6)) belong to the core genome of *Klebsiella* and should not be regarded as acquired. Table N shows the resistance genes found in the *K. pneumoniae* isolates. Several aminoglycoside resistance genes were detected, as well as sulfonamides and trimethoprim resistance genes. Fluoroquinolone resistance was mediated by mutations, plasmid-mediated quinolone resistance genes and the bifunctional *aac(6′)-Ib-cr* gene. Detailed information can be found in Supplementary File S1.

### 2.3. Plasmid Content

Twenty different plasmid replicon types were found, and several of them occurred in different animal species. The most prevalent replicons were ColRNAI (n = 72, found in human and primate isolates) and IncFIB(K) (n = 31, found in human, equine, primate, canine and feline isolates), followed by IncFII(K) (n = 14, found in human, equine, primate, and feline isolates), Col440I (n = 10, found in human isolates only) and IncHI1B (pNDM-Mar) (n = 7, found in human and primate isolates). The other plasmid replicons were carried by four or less isolates, mostly of human origin. Nine isolates did not carry any known plasmid replicons (Supplementary File S1).

### 2.4. Virulence Gene Content

Host-specific differences were also observed in the distribution of virulence genes. In general, the vervet isolates carried more virulence genes, and the virulence genes frequently differed from those present in human isolates (Supplementary File S1). The yersiniabactin gene was detected in 50% (25/50) of the human *K. pneumoniae* isolates, 70% (14/20) of the vervet isolates and two out of the three horse isolates (Supplementary File S1). Interestingly, the types of the yersiniabactin locus and the associated integrative conjugative element (ICE*Kp*) differed between human and animal isolates. Host-specific differences in the yersiniabactin locus were observed in isolates belonging to ST23. Namely, the human isolate carried *ybt1* on ICE*kp*10, while the five vervet isolates consistently harbored *ybt9* on ICE*Kp*3. All ST60 isolates from vervets (n = 9) and horse (n = 1) were associated with *ybt2* on ICE*Kp*1 (Supplementary File S1).

The aerobactin gene *iuc1* was present in 13 isolates, including five vervet and one human ST23 isolate. In addition, the salmochelin gene was present in ST23 and ST60 isolates from humans and animals. The two genes associated with the hypermucoid phenotypes, *rmpA* and *rmpA2*, were found only in ST23 and ST60 isolates and, mainly in vervet ST23 isolates, only one single human ST23 strain carried the *rmpA2_3* gene (Supplementary File S1).

### 2.5. Virulence and Resistance Scores

Kleborate scores provide a rough categorization of isolates based on their virulence and resistance content. Most isolates displayed a low virulence score of 0, meaning no acquired virulence loci (n = 41), or 1 (yersiniabactin only, n = 27). The 14 isolates with the highest virulence scores included a human ST234 strain with score 2 (yersiniabactin and colibactin, and aerobactin) and all 12 vervet ST23 and ST60 strains that had scores of 4. The only isolate with a score of 5 was the human ST23 strain. Surprisingly, no virulence genes were detected in 25 of the 50 clinical *K. pneumoniae* isolates from human patients, and 22 had a low virulence score of 1. Based on resistance gene content, 53 and 29 isolates were scored as level 0 (fewer resistance genes) and level 1 (ESBL-positive), respectively. No isolates belonged to levels 2 (carbapenemase-positive) and 3 (carbapenemase- and colistin-positive).

### 2.6. K. Variicola and K. Quasipneumoniae

No resistance genes were found in the four *K. variicola* isolates, and only four were detected in the five *K. quasipneumoniae*: *bla*_TEM1b_ in the single human isolate, and *aph(3”)-Ib*, *aph(6)-Id* and *sul1* in one of the two horse isolates. None of the *K. quasipneumoniae* isolates carried a known virulence gene and only one primate *K. variicola* isolate carried an *iro*-like gene, as determined by kleborate, however, its functionality is unknown. Plasmid replicons were only found in three *K. quasipneumoniae* (the two equine isolates carried IncFIB(K) and the human isolate additionally carried ColRNAI (pIGMS32), Col(pHAD28) and IncR) and two *K. variicola* isolates (IncFIB(K) in two primate isolates).

## 3. Discussion

This is one of the few studies investigating the genetic diversity of *K. pneumoniae* in the Caribbean region using a WGS-based approach, and the sole study including both human and animal isolates from this geographical region. Since the collapse of the sugar industry, the number of vervets has outgrown the human population (estimated in up to 60,000 units), and these monkeys have gradually made their way down from the mountain forests and into residential areas, increasing the probability of direct and indirect contact with humans and consequently the risk of zoonotic transmission of infectious diseases. Interestingly, these monkeys are a natural reservoir of *K. pneumoniae* [11], which is a cause in these animals of opportunistic infections such as pneumonia, meningitis, peritonitis, cystitis and septicemia. A previous study on the island reported the occurrence of a small disease outbreak caused by *K. pneumoniae*, expressing the hypermucoviscosity phenotype and capsular serotype K2, in a vervet research colony [21]. All the affected monkeys displayed abscesses, mainly in the abdominal cavity, and less frequently in the lungs, cerebellum and skin.

The present study was primarily conceived to assess whether *K. pneumoniae* isolates from vervets on St. Kitts are epidemiologically related to those causing infections in the local human and domestic animal populations. The results show that *K. pneumoniae* isolates from vervets are genetically unrelated to the vast majority of clinical isolates from humans and other animal species. Two genetic lineages, ST60 and ST23, appeared to be most prevalent in vervets. ST60 was isolated from nine vervets and one from abdominal fluid in a horse. Although it was not detected in any human case in this study, this lineage has been sporadically reported in human infections elsewhere [22] and has been associated with suppurative peritonitis in captive monkeys belonging to another species, the gold-handed tamarin (*Saguinus midas midas*) [10]. ST23 was isolated from five vervets and one patient affected by urinary tract infection during the same period. However, the vervet isolates clustered separately from the human isolate in the phylogenetic analysis and differed with regard to the accessory genome. Namely, the human strain had a virulence score of 5, compared to 4 for the vervet strains. Furthermore, the Yersiniabactin gene was different and located on a different ICE. The aerobactin, Salmochelin and *rmpA* genes (causing hypermucoviscosity) were also different, and only these strains had a genotype that caused hypermucoviscosity, though the phenotypes were not assessed. The human strain had an additional colibactin gene that was not present in the vervet strains. The ST23 lineage is one of the best-known hypervirulent *K. pneumoniae* clones and is able to cause severe disease in apparently healthy individuals [23]. Multidrug-resistant hypervirulent variants of ST23 have been reported in China [24,25,26], India [27] and Korea [28]. Capsular serotype K1 isolates belonging to ST23 have been associated with liver abscesses mainly in Southeast Asia, but sporadic cases, often connected with travel or migration, have been reported from USA, Canada, Spain, France, Belgium, Sweden and Denmark [29]. A recent population study revealed a rapid global dissemination of hypervirulent clonal-group 23, which typically possess an integrative, conjugative element ICE*Kp* encoding the siderophore yersiniabactin and genotoxin colibactin [30]. Our analyses show that the vervet ST23 isolates form a distinct monophyletic cluster in the ST23 population (Figure 2). Among the other three STs found in vervets, ST37 was also isolated from two cases of human infection: one urinary tract infection and one case of sepsis. ST37 is a common lineage in humans, frequently associated with ESBL-production, and has been previously found in animals [31,32,33], but in our study they did not carry any genes encoding ESBL. However, vervet and human ST37 isolates from this study clustered separately in the phylogeny and harbored different antimicrobial resistance genes, though all lacked virulence genes (Figure 1). ST1102 and ST2072 were only detected in vervets, but the former ST has been previously reported in humans [22].

Our results corroborate recent research indicating that host and ecological adaptations in *K. pneumoniae* may limit the spread of resistant or virulent clones across humans, animals and the environment [34]. The same study hypothesized sporadic transmission between humans and companion animals based on the occurrence of healthcare-associated clones such as ST307 and other highly virulent strains in these animals. ST307 is a common ST, globally colonizing and infecting both humans and animals [34,35,36,37]. Our study revealed the occurrence of this lineage in one horse, one cat and four human patients. A few host-specific differences were observed in the distribution of resistance, and none of them carried any virulence genes (Figure 1).

Antimicrobial resistance is an important feature of *K. pneumoniae*. The prevalence of resistance genes among human clinical isolates from St. Kitts was generally low compared to other regions of the world [37]. In particular, carbapenem and colistin resistance genes were not detected, which probably reflects the low usage of these antimicrobials at the local hospital (Andrea Williams, personal communication). The only ESBL-encoding gene was *bla*_CTX-M-15,_ which was detected in 25 of the 50 human isolates among multiple lineages (ST307, ST11, St152, ST323, ST39, ST392, ST219 and ST26-1LV). *bla*_CTX-M-15_ also occurred in an equine and a feline strain belonging to ST307. *K. pneumoniae* isolates from vervets did not contain any ESBL-encoding gene and, except for a single ST37 strain, displayed susceptibility to all antimicrobials, which might reflect the absence of usage of antimicrobials in these animals.

Iron-capturing systems are important virulence factors in *K. pneumoniae*. Yersiniabactin, which allows bacterial growth in iron-deprived situations and protects against phagocyte defenses, is generally present in about one third of the clinical isolates and associated with invasive infections [30]. In our study, we found several variants of yersiniabactin in human and animal isolates. Interestingly, its prevalence was higher in the vervet isolates (70%) than in isolates from humans (51%) or other animal species, where only two out of ten isolates had a virulence gene. Apart from yersiniabactin, only few other iron-capturing systems were found. Four vervet isolates carried genes associated with the hypermucoid phenotype, which has been previously described as a prevalent phenotypic trait in strains infecting captive vervets on St. Kitts [21]. It appears that the isolates infecting vervets possess host-specific virulence traits, namely *rmpA*_1, located on KpVP-1, and *rmpA*-11, associated with the ICE*Kp*1.

Several different mobile genetic elements (MGE), associated with the spread of antimicrobial resistance genes and virulence genes, were detected among the *K. pneumoniae* isolates. We could not associate resistance genes and virulence genes to them due to the fragmented nature of Illumina-based draft genomes. Several plasmid incompatibility groups were shared between human and animal isolates (Figure 1), especially IncF. Primate *Klebsiella* isolates carried several plasmids that were not associated with antimicrobial resistance. These plasmids may instead be involved in virulence.

While performing this study, a cross-Caribbean study based on the WGS of 270 human *Klebsiella* isolates was published [2], further enlarging the knowledge of the population structure of this bacterial species in the Caribbean. Many of the most frequent STs observed in that study were also found in our study (ST11, ST23, ST152 and ST307). However, some of the prevalent STs in our study (ST39 and ST219) were not listed among the main STs in the cross-Caribbean study, indicating possible differences in clonal distribution between countries.

*K. quasipneumoniae* and *K. variicola* could only be identified by WGS analysis using Kleborate, as they were not included in our MALDI-TOF MS database. The significance of these species in disease is not yet clear, especially in animals. Antimicrobial resistance was rare and the prevalence of virulence genes was low in these species. A novel *iro* gene was present in a *K. variicola* isolate from a vervet. The significance of this finding remains unclear.

## 4. Materials and Methods

### 4.1. Bacterial Isolates

The strain collection comprised 82 presumptive *K. pneumoniae* isolates from humans (n = 51), vervets (n = 21), horses (n = 5), dogs (n = 4) and a cat (n = 1) on St. Kitts. Human isolates were isolated from consecutive clinical samples submitted to the clinical microbiology laboratory at Joseph N France General Hospital between March 2017 and January 2018, whereas animal isolates from clinical samples were collected by the diagnostic laboratory at Ross University School of Veterinary Medicine over the period of 2011–2018. After initial identification by classical biochemical tests, species identification was confirmed by MALDI-TOF MS (Vitek MS RUO, bioMérieux, Marcy-l’Étoile, France) using Saramis v.3.5 (bioMérieux) for spectra interpretation.

### 4.2. Whole Genome Sequence (WGS) Analysis

Overnight cultures were grown in tryptic soy broth at 37°C with 200 rpm shaking. Genomic DNA was extracted using the DNeasy Blood and Tissue kit (Qiagen). Library preparation was carried out using the Nextera XT kit and paired-end 2 × 250 bp sequencing on a MiSeq, all following standard Illumina protocols (Illumina, Inc., San Diego, California, USA). All raw reads have been deposited in ENA with BioProject ID PRJEB42526.

De novo assembly and MLST were performed in CLC Genomics Workbench v.11.0 using the tools within the Microbial Genomics Module. Various web-based tools, both at the Centre for Genomic Epidemiology (http://www.genomicepidemiology.org/ accessed on 21 September 2020) and Kleborate [38], a pipeline specifically for the genetic characterization of *Klebsiella* whole genomes, were used for species identification, MLST and plasmid replicon typing, as well as to determine the presence and scores of acquired virulence and antimicrobial resistance markers. RAST was used for analysis of plasmid contigs.

To investigate the genetic relatedness of the isolates, a maximum likelihood tree was generated from core genome SNPs. Briefly, sequence reads were mapped against the reference genome of strain FDAARGOS_156 (GenBank accession CP014123) using NASP v.1.0.0 [39]. First, duplicated regions in the reference were removed using NUCmer followed by aligning of the reads using the Burrows–Wheeler Aligner (Li et al., 2009). All positions with < 10-fold coverage or if the variant was present in <90% of the base calls were excluded. The relatedness of the isolates was inferred using IQ-TREE v.2.1.1 to obtain a midpoint rooted phylogeny. Additionally, using NASP, a phylogeny based on the genomics data of different *Klebsiella* species (39) was generated for species identification, as well as a midpoint-rooted maximum likelihood SNP-based phylogeny using FastTree v.2.1.10 with all available *K. pneumoniae* ST23 genomes (n = 80, accessed 9 July 2020) in NCBI’s RefSeq, with available data on host and geography.

## 5. Conclusions

Human and animal *K. pneumoniae* infections on St. Kitts are usually caused by genetically distinct, host-specific lineages. Although some monkey isolates belong to ST23, which is often associated with hypervirulent epidemic clones in other parts of the world, WGS-based analysis showed these to cluster separately, have a distinct virulence gene profile and display lower abundance of antimicrobial resistance genes compared to human clinical isolates. These and other differences indicate that the risk of *K. pneumoniae* transmission between vervets, humans and other animal species appears to be limited by host and ecological barriers

## Data Availability

ENA BioProject ID PRJEB42526.

## Supplementary Materials

The following supporting information can be downloaded at: https://www.mdpi.com/article/10.3390/antibiotics11010042/s1, Supplementary File S1: Phylogeny and accessory genes of *K. pneumoniae*, Supplementary File S2: strain information.
