# Supplementary figures and images for: One Health Genomic Study of Human and Animal Klebsiella pneumoniae Isolated at Diagnostic Laboratories on a Small Caribbean Island
Source: Antibiotics (Basel). 2021 Dec 30;11(1):42. doi: 10.3390/antibiotics11010042 (PMC8772961; doi:10.3390/antibiotics11010042)

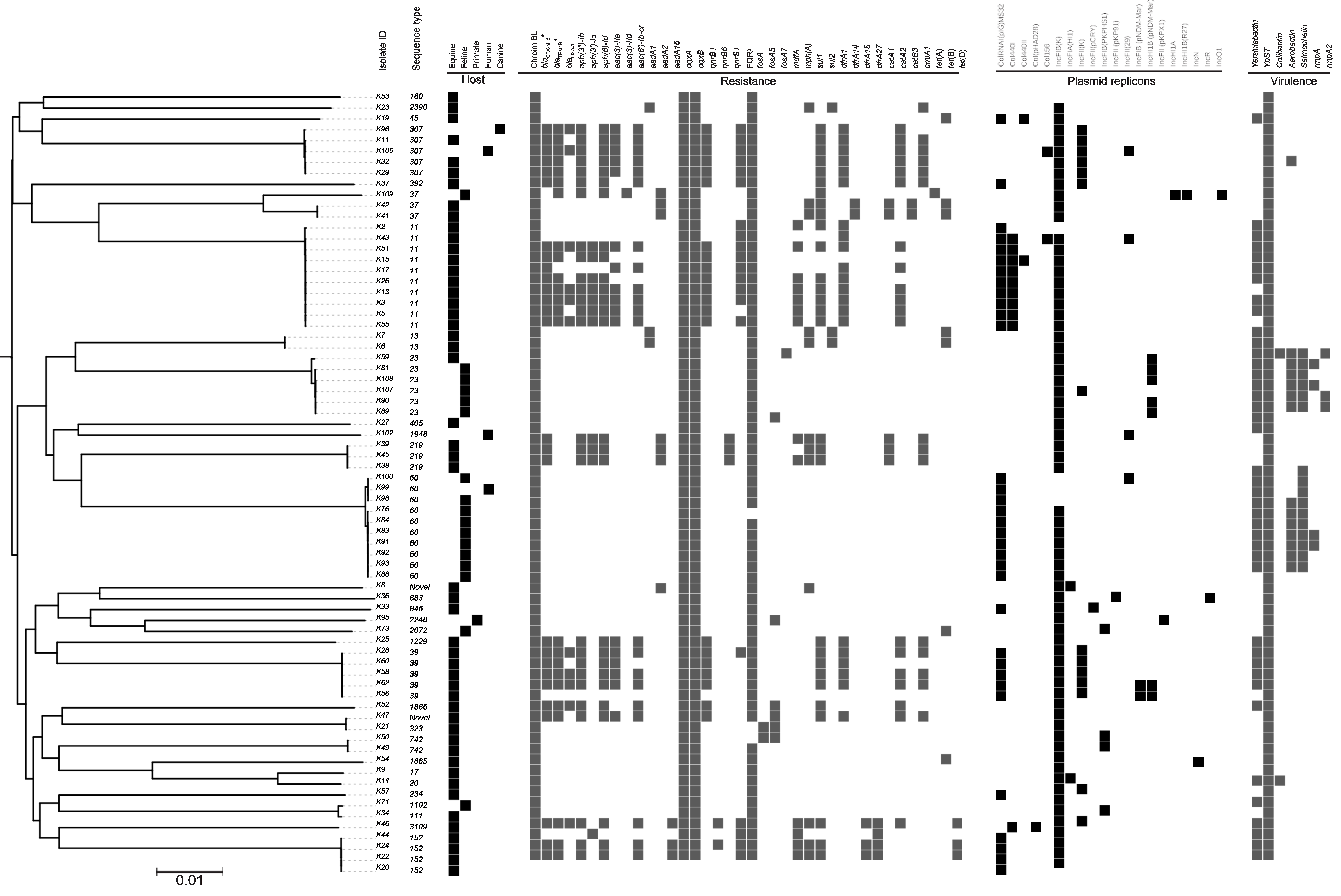

Supplement: Supplementary file 1 [file antibiotics-11-00042-s001.zip › Supplementary File S1.png]
